# Effectiveness of Ayush Rasayana A and B on the Quality of Life of Older Adults: Protocol for a Cluster Randomized Controlled Trial

**DOI:** 10.2196/58186

**Published:** 2024-11-11

**Authors:** Pallavi Mundada, Deepa Makhija, Sunita Mata, Kalpana Kachare, Aparna Manathottathil, Abha Sharma, Bhogavalli Chandra Sekhara Rao, Rakesh Rana, Arunabh Tripathi, Kiran Rana, Vandana Joshi, Ashish Raturi, Anukampa Singh, N Srikanth, Rabinarayan Acharya

**Affiliations:** 1 Central Council for Research in Ayurvedic Sciences Ministry of Ayush Government of India New Delhi India

**Keywords:** Ayush Rasayana A, Ayush Rasayana B, cluster-randomized trial, geriatrics, Ayurveda, quality of life, complementary and alternative medicine

## Abstract

**Background:**

With advancing age among older adults, the associated debilities increase, indicating a deteriorating health status as there is a gradual loss of muscle mass, strength, and functionality. *Ayush Rasayana* A and B are coded Ayurvedic medicines developed from herbal extracts. This study has been planned to prevent debilitating conditions and improve the quality of life (QOL) in older adults.

**Objective:**

This study aimed to assess the effectiveness of *Ayush Rasayana* A and B on the QOL, quality of sleep, and functionality of older adults, along with the tolerability of the intervention.

**Methods:**

This was a multicenter, open-label, cluster randomized controlled trial conducted with 720 participants aged 60 to 75 years. The participants were divided into 2 groups (intervention and control), with both receiving Ayurvedic ancillary treatment for 3 months. The intervention group additionally received 10 g of *Ayush Rasayana* A orally once daily at bedtime for 6 days, followed by 1.5 g of *Ayush Rasayana* B orally twice daily before food for the remaining 84 days. The assessment criteria included the Older People’s Quality of Life Questionnaire Brief, Katz Index of Independence in Activities of Daily Living, Pittsburgh Sleep Quality Index, Five Times Sit-to-Stand Test, and shoulder and scapular movements. Any change in hematological and biochemical parameters and occurrence of treatment-emergent adverse events were also assessed during the study period.

**Results:**

The recruitment of the participants started in December 2023, and the final follow-up was completed in April 2024. Out of the total 720 enrolled participants, 686 (95.3%) completed the study up to the last follow-up.

**Conclusions:**

This study may provide evidence-based data to establish preventive treatment protocols for enhancing the QOL and functionality among older adults. The study results may also be helpful for the planning of interdisciplinary health policies for improving the health conditions of different populations

**International Registered Report Identifier (IRRID):**

DERR1-10.2196/58186

## Introduction

### Background

The aging population in India is a major concern across social, political, and economic spheres. The older adult population in India is growing 3 times faster than the general population, which is attributed to the increased life expectancy [1]. A total of 27.72% of the older adult population reported having a medical condition, either short term or chronic [2]. With advancing age, there is a gradual loss of muscle mass, strength, and functionality. Studies show that chronic morbid conditions such as musculoskeletal disorders impact the quality of life (QOL) in multiple dimensions [3]. The prevalence of age-related diseases is on an upward trend, and its management is limited at this age owing to less reciprocation and multiple underlying disorders [4]. In addition to causing symptoms of structural and functional deficiencies, these age-related illnesses cause a reduction in the older adults’ total functional ability and a variety of limitations. The aging process is brought about by the damage inflicted on biological macromolecules, which act as endogenous damaging agents, creating a state of oxidative stress. This accumulated damage in cells over time leads to the loss of function or functional impairments in the body [5]. Reduced quality of sleep and increased prevalence of sleep disorders are common in advanced age. Studies show that older people have low sleep quality, and it is often associated with a lower QOL [6]. Aging is inevitable, and there is an urgent need to find interventions that help reduce the incidence of debilitating age-related diseases and promote QOL.

Ayurveda endures as a traditional and extensive discipline of traditional Indian medicine used to promote health, prevent illness, and treat physical ailments via *Jarachikitsa* or *Rasayana*. The medicines, food products, and lifestyle-related factors that enhance the quality and longevity of life have been termed *Rasayana* [7]. It is a rejuvenation therapy that has been said to impede the aging process (*Vayasthapanam*), lengthen life expectancy (*Ayushkaram*), improve intelligence (*Medha*), build strength (*Bala*), and prevent disease development [8]. According to various studies, the *Rasayana* drugs possess potent antioxidant activity when combined with dietary supplements and rejuvenators. It reacts negatively with oxidative stressors, which are what lead to the production of various free radicals [9,10]. The study drugs—*Ayush Rasayana* A and B—are coded Ayurvedic medicines; are developed from herbal extracts that have rejuvenating properties as per the classical Ayurveda textbooks; and are effective in improving health-related QOL, functional capacity, and cognition [11]. This study is planned as a multicenter, double-arm, cluster randomized controlled trial to assess the effectiveness of *Ayush Rasayana* A and B on the QOL of older adults in the selected populations.

### Objectives

The primary objective of the study is to assess the effectiveness of *Ayush Rasayana* A and B on the QOL of older adults. The secondary objectives include the assessment of the quality of sleep and functionality in the study population, along with the assessment of the tolerability of the intervention during the study period.

## Methods

### Study Design

The study was an open-label, multicenter, parallel-group, cluster randomized controlled trial conducted in 2 groups with an allocation ratio of 1:1.

### Study Participants

#### Inclusion Criteria

Participants aged 60 to 75 years were selected for the study from the identified Scheduled Caste (SC)– or Scheduled Tribe (ST)–dominant areas regardless of gender. They needed to be ambulatory, be clinically stable with or without medication, have a score of 5 or more in the Katz Index of Independence in Activities of Daily Living, and be willing to provide written informed consent for their participation in the study for 3 months.

#### Exclusion Criteria

Participants with blood pressure ≥160/100 mm Hg, hemoglobin A_1c_ (HbA_1c_) ≥8%, serum thyroid-stimulating hormone >10 mIU/mL with or without medication, elevated liver enzymes (aspartate transferase and/or alanine transaminase >2 times of the upper normal limit), impairment of renal function (defined as serum creatinine>upper normal limit), or BMI <23 or >30 kg/m^2^ were excluded from the study. Those who have a history of serious cardiac dysfunction or pulmonary dysfunction (patients with asthma and chronic obstructive pulmonary disease); those with cancer or chronic inflammatory conditions, including but not limited to chronic infection like tuberculosis, leprosy, HIV, and collagen vascular diseases; participants on hormone replacement therapy, steroid therapy, chemotherapy, or immunosuppressive therapy; and participants with alcohol use disorder were not enrolled. Those with a diagnosis of any severe cognitive or psychiatric illness, neurological disorders, hemorrhagic diseases, or coagulation disorders within 90 days before the screening were also excluded. Participants who were currently on any drugs that affected the cognitive or physical function, on any nutritional supplements or any Ayush or folk medicine for maintaining the QOL, or with any other condition that the investigator thought could jeopardize the study were also excluded from participation.

### Withdrawal Criteria

Study participants who developed any serious adverse events, developed any condition mentioned in the exclusion criteria during the study period, and were not compliant with the protocol or not willing to continue in the study were free to withdraw from the study. Detailed justification for withdrawal of the participant were prepared, indicating the line of further management, if required. The sponsor and ethics committee were informed within the appropriate time as per Ayush Good Clinical Practice guidelines.

### Study Setting

The study was conducted as a part of the public health care program, through 17 peripheral institutes under the Central Council for Research in Ayurvedic Sciences (CCRAS), Ministry of Ayush, Government of India. For the Ayurveda Mobile Health Care Programme under Scheduled Caste Sub Plan, areas predominantly dwelled by SC population were selected by 8 institutes located at Agartala, Gwalior, Patna, Chennai, Vijayawada, Jammu, Guwahati. and Bangalore, while for the Tribal Health Care Research Programme under Tribal Sub Plan, areas predominantly dwelled by ST population were identified by 9 institutes located at Cheruthuruthy, Bangalore, Nagpur, Gangtok, Ahmedabad, Bhubaneshwar, Kolkata, Mumbai, and Jammu. The participants were screened through outreach outpatient departments, medical camps, and door-to-door visits.

### Study Intervention

Participants from both groups (intervention and control) were provided with ancillary care through Ayurveda for 90 days. In the intervention group, along with ancillary care, 10 g of *Ayush Rasayana* A in powdered form were administered orally with lukewarm water, once daily after food at bedtime for the first 6 days starting from the day of enrollment, and 1.5 g of *Ayush Rasayana* B were administered orally per day in 2 divided doses (2 capsules of 375 mg each) with lukewarm water, before food for 84 days (from the 7th day onward up to the 90th day of enrollment).

**Figure 1:**
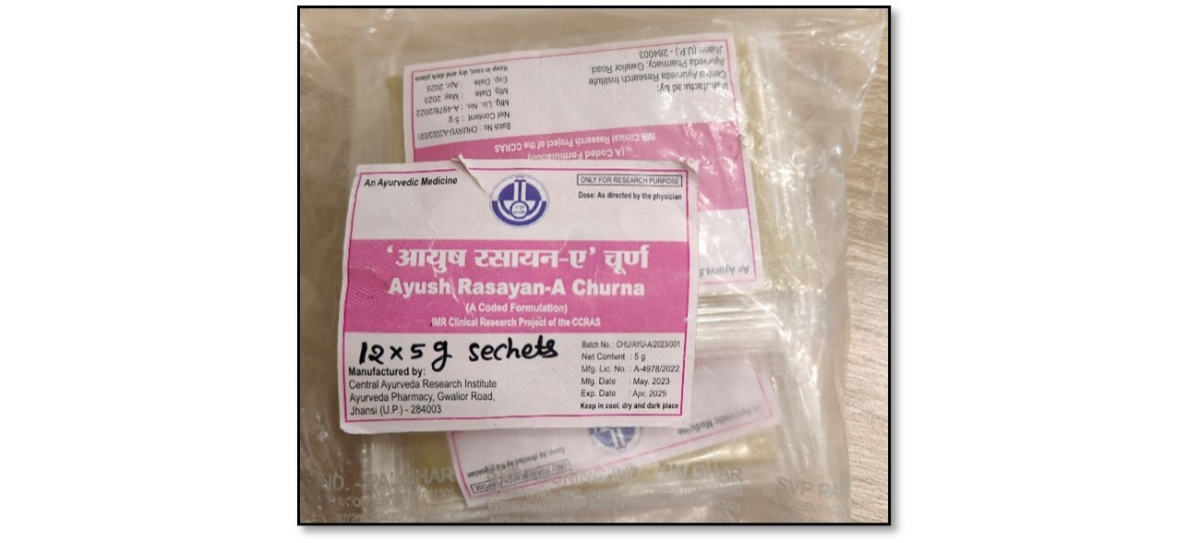
Image of the study intervention: Ayush Rasayana A, procured from the manufacturer.

**Figure 2:**
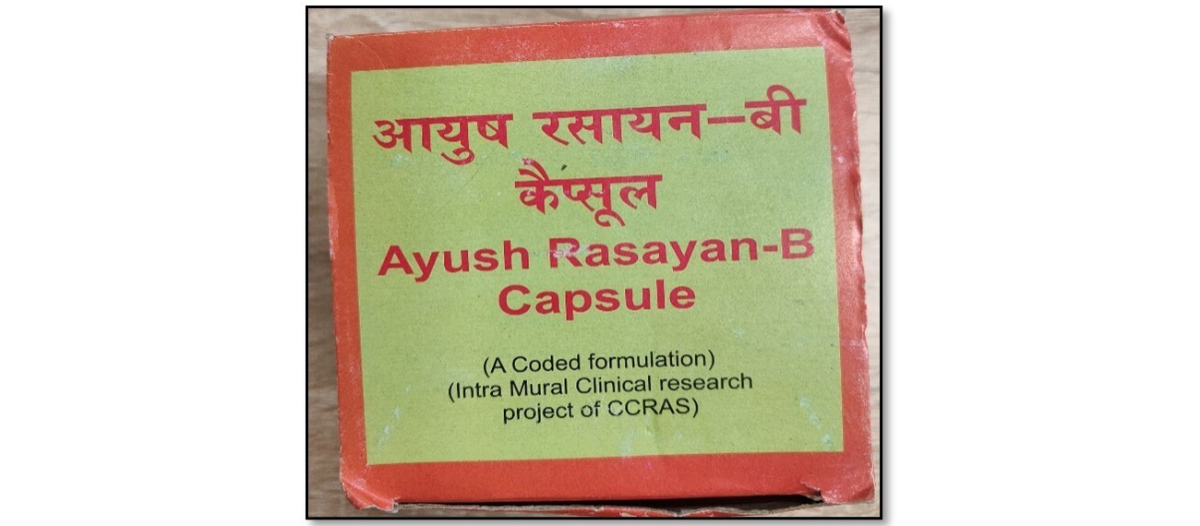
Image of the study intervention: Ayush Rasayana B, procured from the manufacturer.

Ancillary care was defined as the care needed by research participants but not necessary to ensure scientific validity, prevent study-related harms, or address study-related injuries [12]. The medicines for ancillary care were procured from Indian Medicines Pharmaceutical Corporation Limited, Ministry of Ayush, Government of India. The dose and duration of ancillary care medicine were decided by the investigator based on the clinical presentations mentioned in the protocol. The study interventions *Ayush Rasayana* A and B were coded Ayurvedic formulations developed by the CCRAS (Table 1). These drugs were manufactured by the Central Ayurveda Research Institute Ayurveda Pharmacy, Jhansi, Uttar Pradesh, India (Figures 1 and 2).

**Table 1:** Ingredients of the intervention (Ayush Rasayana A and B).

Study drug	Ingredients
*Ayush Rasayana* A	Harītakī (Terminalia chebula Retz) Sanāya (Cassia angustifolia Vahl) Yashtimadhu (Glycyrrhiza glabra Linnaeus) Rock salt
*Ayush Rasayana* B	Amalaki (Phyllanthus emblica Linnaeus) Asvagandha (Withania somnifera [Linnaeus] Dunal) Gudūcī (Tinospora sinensis [Loureiro] Merrill)

### Outcome Measures

The primary outcome measure was the change in the QOL of the older adults, assessed by Older People’s Quality of Life Questionnaire-Brief (OPQOL-BRIEF) at baseline and on the 34th day, 62nd day, and 90th day. The secondary outcome measures included any change in the Katz Index of Activities of Daily Living, Pittsburgh Sleep Quality Index (PSQI), Five Times Sit-to-Stand Test, shoulder joint mobility, hematological and biochemical parameters (complete blood count, liver function test, and renal function test), and the occurrence of treatment-emergent adverse events during the study period at the baseline and on the 90th day.

The OPQOL-Brief is a short measurement for QOL developed from the OPQOL-35 questionnaire [13]. It contains 13 questions scored from 1 (strongly agree) to 5 (strongly disagree) based on the response from the participant. The total score is calculated after reverse coding the positive items so that higher scores represent higher QOL. The Katz Index of Activities of Daily Living is a dichotomous scoring tool used for measuring independence in older adults. It measures the functional independence of a person, assessing self-care tasks under 6 domains: bathing, dressing, toileting, transferring, continence, and feeding [14]. The PSQI is a widely used scale comprising a combination of Likert-type and open-ended questions in 7 components: subjective sleep quality, sleep latency, sleep duration, sleep efficiency, sleep disturbance, use of sleep medication, and daytime dysfunction—each with scores ranging from 0 to 3. Higher PSQI scores indicate more acute sleep disturbances, thus a lower quality of sleep [15]. The Five Times Sit-to-Stand Test is a measure used to assess functional independence and mobility. It evaluates the time required to stand as rapidly as possible from a sitting posture for 5 repetitions; the shorter the time taken by the person, the better the functional condition [16]. The change in shoulder joint mobility is assessed through simple physical maneuvers for the active range of movements: active abduction, and external and internal rotations. Each maneuver is assessed separately by 3-point scales for the range of mobility (fully able=3, partially able=2, and unable=1) and pain on movement (no pain=1, some pain=2, and lots of pain=3) on both sides. A sum score for all 3 maneuvers on each side is estimated for the range of movement and pain separately.

**Figure 3:**
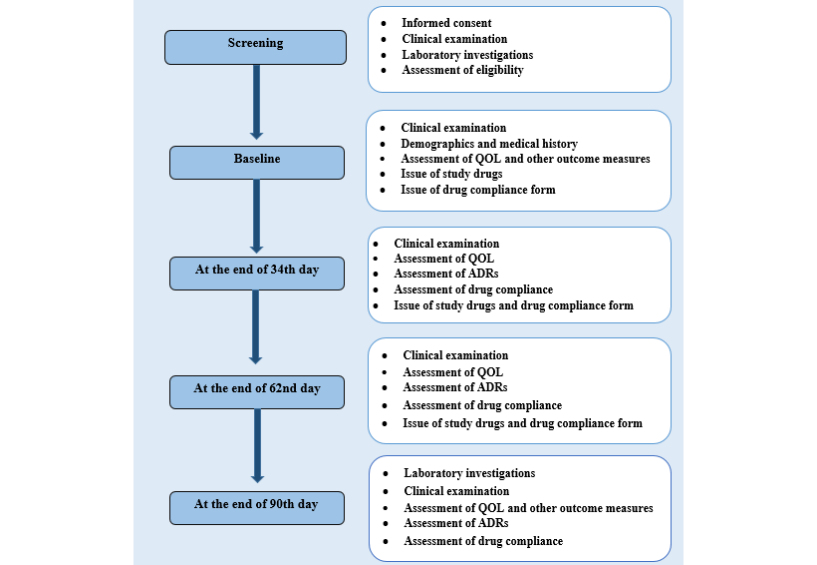
Flow diagram depicting the study schedule for the assessment of effectiveness of Ayurvedic intervention in the QOL of older adults through the cluster randomized controlled trial. ADR: adverse drug reaction; QOL: quality of life.

**Figure 4:**
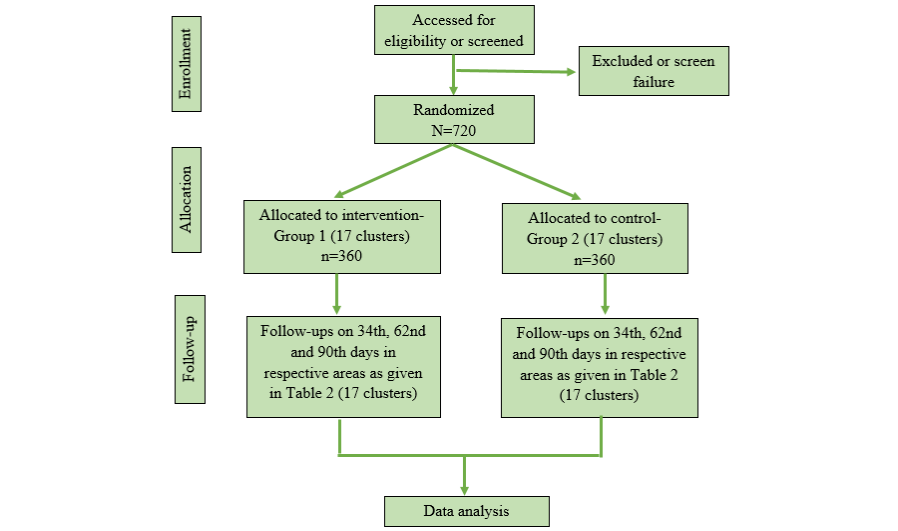
CONSORT (Consolidated Standards of Reporting Trials) diagram depicting the enrollment, allocation, and follow-up visits for the assessment of QOL in older adults through the cluster randomized controlled trial. QOL: quality of life.

The schedule of enrollment, administration of study intervention, assessments, and follow-up visits are shown in Figures 3 and 4.

### Sample Size

A change of 9 points on the OPQOL-Brief was expected in the intervention group (*Ayush Rasayana* A and B) compared to 6 points in the control group. With an SD of 8 points and a 95% CI, a sample of 150 participants per group would be needed to achieve 90% power. The estimates for the intervention group and SD were based on the results of a previously published study [11]. Considering the design effect of 2 clusters in this randomized controlled trial and an attrition rate of 20%, a sample size of 360 participants per group was needed. The sample size of each center needed to be 20 in each of the 2 groups. Therefore, the total sample size for each group was calculated as 360 for 18 study centers, and for the 2 groups, the total sample size was 720 (360 × 2). Therefore, a total of 720 participants were intended to be enrolled in the study. Initially, the sample size was calculated for 18 centers, and later on, 1 center was withdrawn from the study and the remaining sample size was distributed to the other centers as per Table 2.

**Table 2:** Sample size distribution at 17 study centers for the evaluation of Ayush Rasayana A and B in older adults.

Area and location of the center		Sample size, n
**Scheduled Tribe–dominated areas (8 centers and 16 clusters)**		
	Agartala	44^a^
	Gwalior	44^a^
	Patna	44^a^
	Chennai	44^a^
	Vijayawada	44^a^
	Jammu	40^b^
	Guwahati	40^b^
	Bangalore	40^b^
	Total	340
**Scheduled Caste–dominated areas (9 centers and 18 clusters)**		
	Cheruthuruthy	44^a^
	Bangalore	44^a^
	Nagpur	44^a^
	Gangtok	44^a^
	Ahmadabad	44^a^
	Bhubaneswar	40^b^
	Kolkata	40^b^
	Mumbai	40^b^
	Jammu	40^b^
	Total	380

^a^Clusters with a sample size of 22 in each group.

^b^Clusters with a sample size of 20 in each group.

### Randomization

The preexisting groups of the population in the selected areas were identified and divided into 5 clusters at each center as per the geographical demarcation. Of these, 2 clusters were randomly assigned to either the intervention or control group by a cluster randomization chart, prepared using a computer-generated sequence by the statistics department of the CCRAS. The participants were recruited through door-to-door surveys, outreach outpatient departments, and medical camps from the selected clusters by the investigators at each study center using voluntary sampling.

### Blinding

The study was open label in execution; therefore, blinding was not done.

### Data Collection

The investigators complied with the requirements for all assessments and data collection. The demographic data, clinical history, details of concomitant medications, and outcome parameters were documented. During follow-ups, the occurrence of any symptom and need for any rescue medication were recorded**.** Data capture for this study was planned to be in both hard copies of the case report form (CRF) and an electronic format. All source documentation supporting entries into the CRFs was maintained, and data were checked for consistency, omissions, and any apparent discrepancies. After the completion of the study, the data will be analyzed and published without disclosing the personal identification of the participants.

### Concomitant and Rescue Medication

The drug or medications administered to treat the participant’s additional illness were noted by the study investigators. The participant continued any concomitant therapy for diabetes mellitus, hypertension, or any other disease during the study, which were not specifically excluded. Any medical emergency would have been relieved with the administration of any rescue drug. This would have been properly documented in the CRF.

### Statistical Analysis

After data collection, verification of its accuracy and limits will be done. The filtered data will be utilized for additional analysis and interpretation. Categorical variables (eg, bowel movement: regular or irregular) will be reported as number (percentage), and the analysis of the pretrial-posttrial outcome will be done by a McNemar chi-square test. Continuous variables (eg, score and laboratory parameter) will be analyzed by a 2-tailed paired *t* test or Wilcoxon test as per the distribution of data in the pretrial-posttrial situation. Categorical variables will be analyzed through a chi-square test in the between-group comparison. Continuous variables will be analyzed through a 2-tailed unpaired *t* test or Mann Whitney *U* test in the between-group comparison. The assessment parameters assigned in more than 2 follow-ups will be analyzed using the r-ANOVA, Friedman test, or Cochran *Q* test. Continuous data with normal distribution will be represented as mean (SD), and data without normal distribution will be represented as median (IQR). The 5% level of significance will be used throughout the analysis. The SPSS (version 26.0l IBM Corp) software will be used to conduct the analysis.

### Monitoring

To ensure strict adherence to the study protocol and correct documentation of the data, CCRAS headquarters monitored the progress of the study. The CCRAS representatives and regulatory authority monitors visited the investigators to inspect the facilities and various records of the clinical study (eg, CRFs and other pertinent data), ensuring that participant confidentiality is respected. The Clinical Monitoring Committee was responsible for verifying the CRFs at regular intervals throughout the study to verify adherence to the protocol; completeness, accuracy, and consistency of the data; and adherence to local regulations on the conduct of clinical research. Any problems detected in the course of these monitoring visits, including delays in completing CRFs, were resolved by the committee.

### Ethical Considerations

The study was conducted according to the national ethical guidelines for biomedical and health research involving human participants (2017); Indian Council of Medical Research; and Good Clinical Practice guidelines for Clinical Trials in Ayurveda, Siddha, and Unani Medicine, 2013. The recruitment started after obtaining approval from Institutional Ethical Committees from all the study centers (the approval details for all 17 centers are shown in Multimedia Appendix 1) and registration in Clinical Trial Registry-India (CTRI/2023/06/054204; dated June 20, 2023). Before initiation, local authorities of the selected sites were informed about the program and the study. The written informed consent for their participation in the study was obtained from each participant after explaining the study in detail in their regional language. The privacy and confidentiality of every participant will be protected, and all the data will be stored in password-protected Microsoft Excel formats accessible to the authorized personnel only. All study participants were insured under Clinical Trial Insurance CTI policy, which covered any adverse events that may occur during the study.

### Protocol Amendments

Any modification from the study protocol were executed after prior approval from the Institutional Ethical Committees.

## Results

The recruitment of the participants started in December 2023, and the final follow-up was completed in April 2024. Out of the total 760 enrolled participants, 686 (95.3%) completed the study up to the last follow-up. The study findings will be disseminated in peer-reviewed journals after the completion of data analysis.

## Discussion

### Expected Findings

The proposed study will evaluate the effectiveness of *Ayush Rasayana* in improving the QOL, quality of sleep, and functionality of older adults. Previous research [11] conducted on healthy volunteers between the ages of 60 and 75 years has shown that these interventions are likely to be effective in enhancing the physical endurance, QOL, and cognition of older adults, thus improving their general well-being. The study used the World Health Organization QOL-BREF questionnaire and the 6-minute walk distance as efficacy measures for 6 months. The study suggests a beneficial effect of the drugs on the aging process [11]. This study is being conducted to substantiate the effectiveness and realize the full potential of the intervention in comparison with a control group.

*Ayush Rasayana* A contains 3 herbal ingredients—*Harītakī* (*Terminalia chebula* Retz), *Sanāya* *(Cassia angustifolia* Vahl), and *Yashtimadhu* (*Glycyrrhiza glabra* Linnaeus)—that are described as having mild laxative properties. Systemic biocleansing or *Samsodhana* is one of the prerequisites for the *Rasayana* procedure as per Ayurvedic textbooks [17]. The intake of the laxative drugs for 6 days before administering the *Ayush Rasayana* B drug will help purify the body and correct digestion and absorption.

After this, *Ayush Rasayana* B, containing the *Rasayana* drugs such as *Asvagandha (Withania somnifera* [Linnaeus] Dunal), *Amalaki (Phyllanthus emblica* Linnaeus), and *Gudūcī* (*Tinospora sinensis* [Loureiro] Merrill), is administered. *Asvagandha* has been proven to have antistress, neuroprotective, antitumor, antiarthritic, analgesic, and anti-inflammatory properties and is useful in different clinical conditions such as dementia, memory loss, stress-induced diseases, Parkinson diseases, etc [18]. *Amalaki* is one of the important rejuvenator drugs, either alone or as a chief ingredient in many polyherbal formulations, mentioned in Ayurveda classics. The consumption of *Amalaki* have been proven to be beneficial in age-related health conditions owing to its immunomodulatory, antioxidant, cytoprotective, antitussive, and gastroprotective properties. In vivo and in vitro studies conducted on *Amalaki Rasayana* have demonstrated improved exercise tolerance and significant improvement in fatigue time in treadmill exercise in aging rats [19]. The results from a study of *Amalaki Rasayana* on healthy volunteers suggest that it may prevent erosion of telomeres in older adults and promote healthy aging [20]. *Gudūcī* is one of the widely used medicinal plants in Ayurvedic formulations, which is reported to have antioxidant, antistress, hepatoprotective, immunomodulatory, anti-inflammatory, antiarthritic, antidiabetic, antiperiodic, antispasmodic, antiallergic, antineoplastic properties, etc [21] The beneficial effects of *Gudūcī* in reducing stress-induced damage in aging cells have been demonstrated in in vivo studies [22]. *Gudūcī* is one of the drugs mentioned as effective in improving intellect and cognition. The aqueous extract of *Gudūcī* significantly improved memory status in rats along with reducing oxidative stress, indicating its beneficial effect on age-related memory and cognitive functions [23]. Thus, a combination of these 3 herbal drugs in *Ayush Rasayana* B is expected to bring about improved QOL in older adults if provided after proper purification using *Ayush Rasayana* A.

### Limitations of the Study

This was a multicenter clinical study focused on older adults. The research was conducted within the framework of a public health program, which constrained the opportunity for detailed, specific laboratory investigations. The duration of intervention was short, and follow-ups without intervention were not included in the study design.

### Conclusion

The administration of *Ayush Rasayana* A and B has the potential to improve older adults’ QOL owing to the proven *Rasayana* properties of the ingredients. This study will help provide evidence-based data to establish preventive treatment protocols for enhancing the QOL and functionality among older adults. The results of the study could be beneficial for the development of public health programs in the older adult population. The study results may also be helpful for the planning of interdisciplinary health policies to improve the health conditions of different populations.

## Appendix

Multimedia Appendix 1 Institutional Ethical Committee (IEC) approval details of the study centers.

## Abbreviations

CCRAS: Central Council for Research in Ayurvedic Sciences
CRF: case record form
HbA1c: hemoglobin A1c
OPQOL-BRIEF: Older People’s Quality of Life Questionnaire-Brief
PSQI: Pittsburgh Sleep Quality Index
QOL: quality of life
SC: Scheduled Caste
ST: Scheduled Tribe

## Data Availability

Data maintained by the central biostatistical monitoring team of the council will be made available in accordance with ethical guidelines for biomedical research and provisions of the council, as feasible.
