# Supplementary material for: Effectiveness of Ayush Rasayana A and B on the Quality of Life of Older Adults: Protocol for a Cluster Randomized Controlled Trial
Source: JMIR Res Protoc. 2024 Nov 11;13:e58186. doi: 10.2196/58186 (PMC11589494; doi:10.2196/58186)
Supplement: Multimedia Appendix 1 [file resprot_v13i1e58186_app1.docx]

**IEC approval details of the study centres**

| **Scheduled Tribe–dominated areas** | | |
| --- | --- | --- |
| 1 | Agartala | 1-34/2023-24/RARC-AGT/IEC/151 dated 22/05/2023 |
| 2 | Gwalior | 5-5/2021-22/ क्षे.आ.अ.सं.-ग्वा./तक./आई.ई.सी./648 dated 04/08/2023 |
| 3 | Patna | 15-1/RARI/Patna/2022-23/IEC/P.F./133 dated 06/05/2023 |
| 4 | Chennai | 4/1/2023/ALRARI/IEC/59 dated 29/04/2023 dated 29/04/2023 |
| 5 | Vijayawada | 4-22/2022-23-RARI/VJA/Tech-IEC/181 dated 03/05/2023 |
| 6 | Jammu | 5-3/2023-RARI/THCRP//Tech/73 dated 26/04/2023 |
| 7 | Guwahati | 2-2/2023-CARI/Gty/Admin/507 dated 24/05/2023 |
| 8 | Bangalore | 6-5/CARI/BNG/IEC Meet/2020-21/ 218 dated 22/05/2023 |
| **Scheduled Caste–dominated areas** | | |
| 1 | Cheruthuruthy | 8/16/2023/NARIP/Tech/meeting/179 dated 03/05/2023 |
| 2 | Bangalore | 6-5/CARI/BNG/IEC meet/2020-21/219 dated 02/05/2023 |
| 3 | Nagpur | 4-6/2022-23/RARI-NGP/IEC/Tech/180 dated 17/05/2023 |
| 4 | Gangtok | 6-10/2007/RARI-GTK/Tech/153 dated 12/05/2023 |
| 5 | Ahmadabad | 4/1/2023-24/क्षे.आ.अ. सं. /अहम /तक. /11 dated 08/05/2023 |
| 6 | Bhubaneswar | 6-199/2016-CARI/BBSR/Tech/162 dated 16/05/2023 |
| 7 | Kolkata | 2-86/2018-CARIDD/Admn/SCSP(Vol-IX)/209 dated 02/05/2023 |
| 8 | Mumbai | 6-94(IEC)/2023-24/Tech/Mum/602 dated 03/07/2023 |
| 9 | Jammu | 5-2/2023-RARI/SCSP/Tech/2 dated 26/04/23 |
